# An inexpensive system for imaging the contents of multi-well plates

**DOI:** 10.1107/S2053230X18016515

**Published:** 2018-11-29

**Authors:** Andrew Bohm

**Affiliations:** a Department of Developmental, Molecular and Chemical Biology, Tufts University School of Medicine, 136 Harrison Avenue, Boston, MA 02111, USA

**Keywords:** automated microscope, imaging system, multi-well plates

## Abstract

An inexpensive system for automated imaging of the contents of 12-, 24- and 96-well plates has been built. The *xyz* stage is constructed from parts from a light-duty computer numerical control wood-carving/engraving machine, and the Arduino-based board was wired so that it can trigger still images or movies though a microscope-mounted digital camera. The translation stage provides reproducible three-dimensional movement of the sample over a volume of 160 mm in *x*, 100 mm in *y* and 40 mm in *z*. A Python script generates the G-code command file that scans the plate and collects a series of *z*-stacked images of each sample. A second Python script automates the calculation of images with a digitally enhanced depth of field. The imaging system is currently being used to facilitate screening for protein crystals, but it could be used to automate the imaging of many other types of samples in multi-well plates.

## Abstract 2

This paper describes the construction of a low-cost, open-source system for imaging the contents of multi-well plates.

## 1. Introduction   

In biology, as well as other fields, many image-based experiments are conducted in multi-well plates, and automated systems for imaging the contents of such plates have been commercially available for many years. These systems are particularly useful in the field of protein crystallography, where hundreds of crystallization experiments are routinely conducted on each new protein construct (Pusey *et al.*, 2005 ▸). Despite their obvious utility, automated imaging systems are prohibitively expensive for many laboratories. Thus, while such systems are common in industrial settings, core laboratories and structural genomics centers, they are not often seen in individual academic laboratories. This is unfortunate because these devices not only increase the number of crystallization trials that each scientist can manage, but also provide a permanent record of the multitude of crystallization experiments such laboratories routinely conduct. Here, we describe the construction and use of an inexpensive crystal-imaging system. While at least one open-source 3D microscope has been described (Wijnen *et al.*, 2016 ▸), to our knowledge this is the first such instrument suitable for imaging multi-well plates. In addition to crystals, this system can be used to image cells and other samples, and it should be of utility in many contexts.

## 2. Construction   

**Figure 1:**
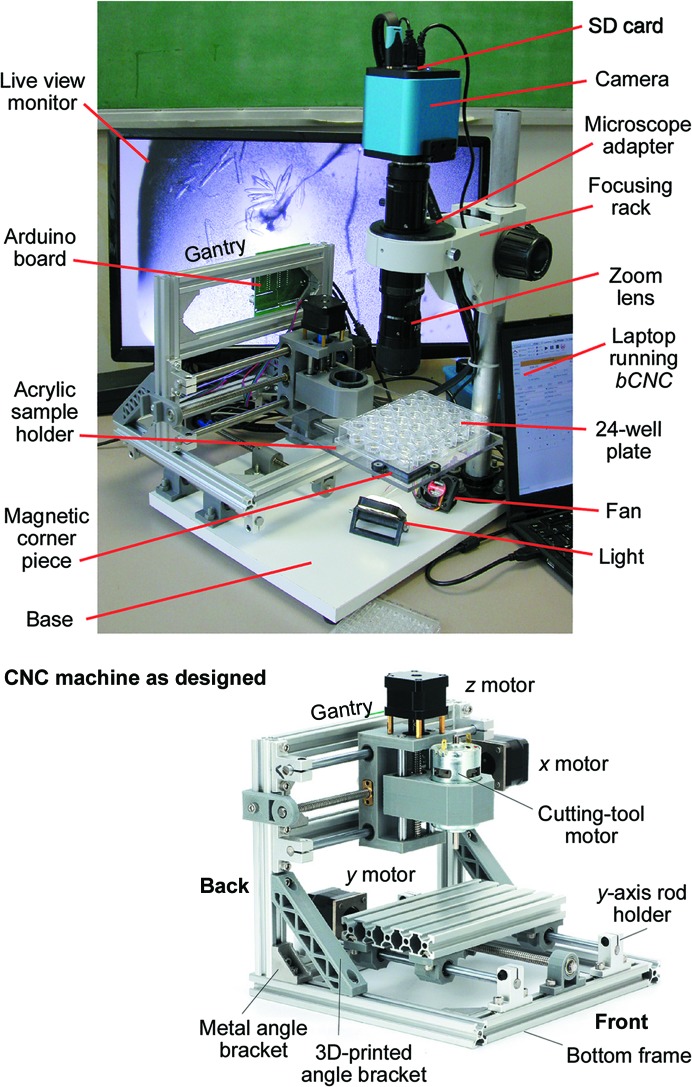
Top: image of the completed imaging system with the key parts labeled. Bottom: the completed CNC machine kit when assembled following the manufacturer’s instructions.

The microscope system is built around an inexpensive computer numerical control (CNC) engraving/wood-carving machine, which was repurposed as a movable *xyz* translation stage. As originally designed, the CNC machine allowed computer-controlled translation in *x* and *z* of a rotary cutting tool, and the object being carved was translated in *y*. The engraving machine is driven by an Arduino-compatible ‘Woodpecker CNC’ circuit board running *GRBL*. (Other circuit boards of this type should work equally well, although users will need to use Arduino software to provide the board with information such as the number of steps per turn of the motors and the pitch of the translation screws.) Simply mounting a microscope in place of the cutting tool was not practical because of the weight of the optics and the vibration during movement. Thus, we chose to move only the multi-well plate and to keep the microscope fixed. To achieve this, we rearranged the parts of the CNC machine so that the entire machine is translated in *y* (Fig. 1 ▸). Additional details and construction notes are provided in the supporting information. The reconfigured translation stage was screwed to a laminated wooden base and the video-microscope system was also mounted on this base.

A sample holder capable of securely holding 12-, 24- and 96-well plates was constructed from 1/4 inch clear acrylic and 3D-printed parts (see the supporting information for details and 3D printer files). The position of the top right corner of the multi-well plate is fixed by a small, printed edge piece. Neodymium magnets above and below the acrylic allow easy adjustment of the bracket that holds the opposite corner of the multi-well plate. The sample holder is completely adjustable; it can securely hold nonstandard sized plates and even round petri dishes.

Optimal imaging depends on both the angle and the position of the light source. We experimented with a variety of different light sources, including a conventional 150 W halogen microscope source equipped with fiber optics and a curved mirror. When correctly positioned, we found that a 1 W pure white, 12 V LED with an improvised diffuser yielded images of crystal screening droplets that were at least as clear as those obtained with the much more powerful (and costly) lamp. (A larger lamp still provides the clearest images when the sample area is larger than ∼2 mm.) A 3D-printed piece which holds the 1 W LED and diffuser allows the angle of the light to be adjusted. Again, details are provided in the supporting information. In our experience, an angle of ∼25° from horizontal generally yields the clearest images. To dissipate the heat, a small 12 V computer fan was mounted on a 3D-printed base and used to circulate the air below the sample. The fan is probably only needed when using a larger lamp.

3D printers and CNC machines use a text-based language known as G-code to control their movements. In our system, a G-code script controls the sample movement beneath the microscope and triggers the acquisition of a series of *z*-spaced images of each crystallization droplet. Taking multiple images of each sample allows the depth of field to be digitally enhanced and it alleviates the need for the distance between the sample and the lens to be precisely correct for each sample droplet.

To control the camera shutter, a 12 V relay (Omron G5LE-14 DC12) was attached to the connector that powered the cutting tool in the original CNC machine. (Other 12 V relays should work equally well.) Using a relay allows the camera to be electrically isolated from the control board. G-code command m3, which turns on the cutting tool, now closes the relay. G-code command m5, which turns the cutting tool off, now opens the relay. If a second computer-controlled switch is needed, one can also use switched headers labeled A3 on the control board (see the supporting information for further details).

**Figure 2:**
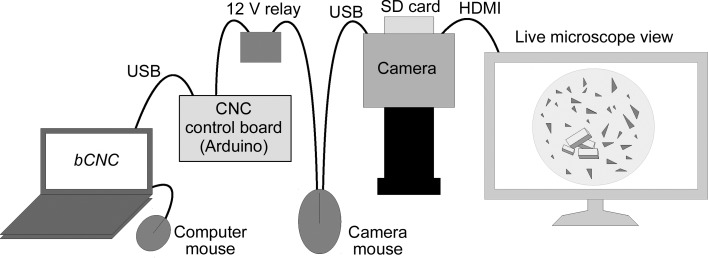
Key connections between the component parts. Note that the mouse connected to the camera has two wires leaving it and that this modified mouse is distinct from the mouse connected to the computer.

On the particular camera we purchased (detailed below), photographs were taken by clicking a USB mouse that was connected directly to the camera. To automate the mouse clicking, we soldered wires to the terminals of the left mouse click switch within the mouse (Supplementary Fig. S2). These wires were connected to the relay. The various connections are shown schematically in Fig. 2 ▸. As discussed below, we were also able to control the shutter of an Olympus E-PM2 Micro Four Thirds format camera using the computer-controlled relay. In this case we modified a hand-held camera shutter switch so that it could be controlled by the solenoid (see the supporting information).

## 3. Cost   

As detailed in Table 1 ▸, the total cost of materials came to approximately $3400. Most of the cost (∼$2500) was for the microscope optics. The translation stage also requires a com­puter to control it. The demands on the CPU are minimal, and virtually any laptop or desktop will suffice. Once the images have been collected the SD card containing them is transferred to a second, faster computer for processing and viewing.

**Figure 3:**
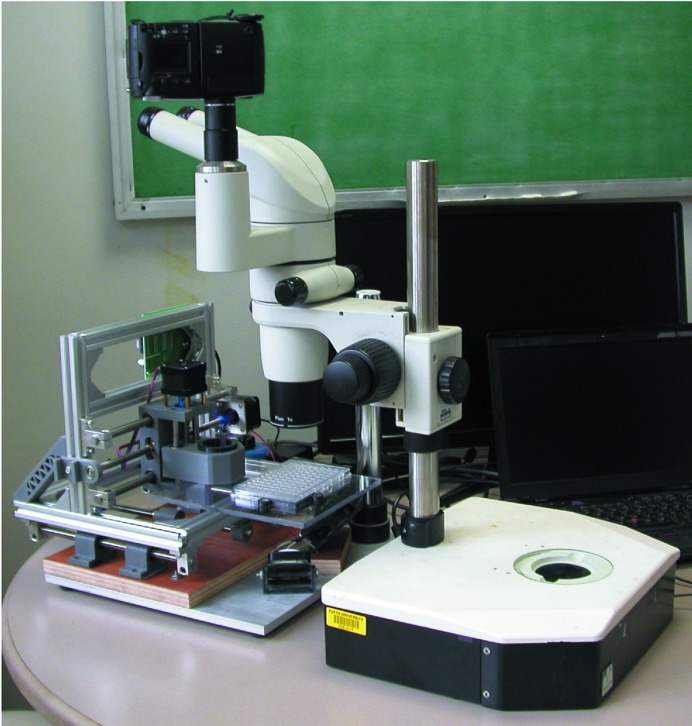
The translation stage can also be used with an existing microscope.

In many cases, it will be possible to use the movable stage described here with an existing microscope. To keep the system as compact as possible, we opted to buy new optics and a new focusing rack. As shown in Fig. 1 ▸, the metal post that supports the microscope was screwed to the base on the right side of the device. To use an existing microscope, one can either remove the post and attach it to the base of the automated system or leave the microscope attached to its lighted base and simply place it in front of the device, with the optics oriented such that they point away from the base, as shown in Fig. 3 ▸. Some microscopes may tip when the optics are in this configuration; placing a heavy object on the base may prevent this.

## 4. Alignment   

To scan a plate, the system must calculate the predicted position of each sample droplet. Rather than try to physically align the plate-holder and translation axes within tens of micrometres, we established a software-based calibration procedure. The plate is first manually translated (using software such as the open-source program *bCNC*) so that the top left drop is centered and in focus. The *x*, *y* and *z* positions are then set to zero via the software. The plate is then translated using the software such that the drops in each of the remaining three corners are centered and in focus, and the *xyz* coordinates are noted. These three sets of coordinates are input to a control file (see the supporting information) along with the number of wells in *x* and *y* on the plate and the number and *z*-spacing of the images to be collected. A Python script writes the G-code commands that will scan the plate and collect the images.

Since three points are sufficient to define a plane, we initially implemented a procedure based on only two coordinates and the origin. This proved to be less accurate than an algorithm using all four corner points. Using a curved mathematical model reliably and reproducibly corrects for nonlinear movements of the device. These nonlinear shifts cause errors that place the sample out of focus if the nonlinear correction is not applied. The mathematical correction based on the four corner drop positions is described in the supporting information.

The G-code sending program *bCNC* has a set of six programmable buttons under the ‘Control’ tab. We found it convenient to program these buttons such that they drive the plate to the approximate position of the corner drops. For instance, the command g0 × 100.15 y -1.34 z 0.13 will move the machine to the specified coordinates. With each corner drop in view, small adjustments are made using the arrow keys in the control window. In our experience, it is usually not necessary to recalibrate between plates made by the same manufacturer. However, re-zeroing when focused on the top right drop will likely be necessary if the machine power is interrupted. Recalibration may also be necessary if the machine is accidentally driven beyond its mechanical limits. This causes a grinding noise, but does not cause any serious damage. (Driving the machine beyond its limits may cause the couplings between the motors and the screws to loosen; these should be checked and tightened before the machine is re­calibrated.)

## 5. Optics, image acquisition and image processing   

The optics used in this system consist of a 12× zoom lens and a 1× video adapter (Thorlabs). This lens ranges from 0.58× to 7× magnification. (As discussed below, a lens system with a smaller zoom range will suffice for most users.) A 3D-printed microscope holder piece (shown in Fig. 1 ▸) attaches the video-microscope optics and camera to a standard focusing rack. Note that the microscope does not pass through the center of the microscope holder. It is off-center to allow the extra space between the point of focus and the metal post that supports the microscope.

For imaging crystallization drops, we use an Amscope 1080p HDMI camera with a 5.5 × 3.4 mm detector and zoom setting between 2× and 3×. At 1× magnification the field of view is the same as that of the detector chip in the camera. At 3× the field of view is one third of these values. Since one rarely uses higher magnification (the field of view is too small), a zoom lens with a smaller magnification range (*i.e.* Thorlabs MVL6X12Z) would have been sufficient and less costly. We could also have reduced the cost of the system somewhat by purchasing a camera that provides less resolution in video mode while retaining the same resolution for still images (*i.e.* Amscope HD102-M). The particular camera we purchased writes images to an SD card, which is manually removed and then mounted on another computer prior to image processing. Since the multi-well plates are manually put on and taken off the imaging system, inserting and removing the SD card requires only a small amount of additional effort.

We also experimented with an Olympus E-PM2 camera. This is a consumer-grade Micro Four Thirds format camera that was connected to the microscope optics via an inexpensive C-mount adapter. The Olympus camera has a much larger detector (21.6 × 13 mm) than the Amscope camera, and it ­has significantly more pixels: 16 megapixels compared with 2 megapixels. As shown in Supplementary Fig. S1(*a*), there is a small amount of vignetting (cropping at the corners of the image) at very low magnification. However, vignetting is completely absent at higher magnification. There is little advantage to using a detector larger than the Micro Four Thirds format, since only the center of the image will be illuminated by the lens. At any magnification, the Olympus camera provided a field of view ∼3.8 times larger in each dimension than that of the Amscope camera. We expected that this would translate into better images, since the sample could be viewed at 3.8 times higher magnification with the same field of view. However, the mechanical shutter on the Olympus causes the microscope to shake slightly, blurring the images at very high magnification. This was not the case with the Amscope camera. Using a Micro Four Thirds format camera with a feature known as ‘electronic front curtain shutter’ or a more costly scientific-grade camera with a larger format detector should solve the camera-shake problem. Additional notes on our experience using the larger format camera are provided in the supporting information.

Regardless of which camera one uses, it is critical that time delays are introduced into the G-code controlling the movement of the multiwell plate and image acquisition. We found that delays of 0.1 s (G-code command g4 p 0.1) are sufficient to allow the system to settle down after sample translations. Additional delays (typically 0.4–0.6 s per image) were introduced to allow the camera sufficient time to write each image. Without these additional delays many images will be skipped. Indeed, even with these delays images are very occasionally missed. To circumvent this problem, we have implemented a procedure whereby, in addition to the data-containing images (*i.e.* five images spaced in *z* by 0.15 mm for 2 µl drops), we also collect one image that is obviously out of focus. JPG compression causes this unfocussed image to be smaller in size than those that flank it, and we have written a Python script, which is included in the supporting information, that uses these poorly focused images to reliably determine the beginning and end of each *z*-stack, even if some images are missing. As currently configured, it takes 13 min to acquire the 576 images for a 96-well crystallization plate. Image-comparison software would likely alleviate the need for the out-of-focus images, but this has not been implemented and it would only reduce the plate-scanning time by about a minute.

**Figure 4:**
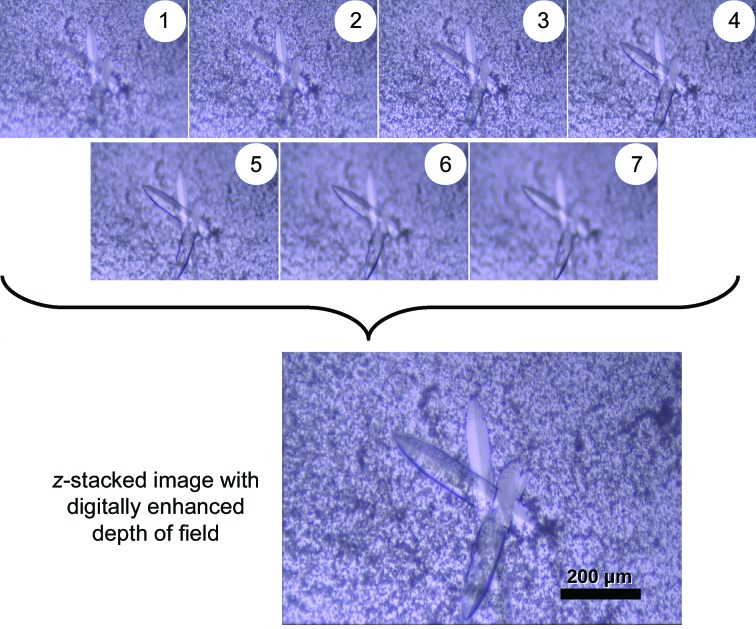
Seven images separated by 50 µm were used to generate a single output image with digitally enhanced depth of field. The images were taken at 5× magnification. The drop shown is a hanging drop that had an initial volume of 5 µl.

The Python script that identifies the start and end of each *z*-stack generates a command file that first aligns the appropriate images and then uses the open-source program *Enfuse* to combine these images into a single, digitally enhanced micrograph. Processing 576 images for a 96-well plate takes approximately 8 min on a 3.5 GHz Core i7 desktop. In our experience, features in the enhanced micrograph are as clear, but not clearer than, those in the parent images. An example of the parent and *z*-stacked images at 5× magnification is shown in Fig. 4 ▸. For the routine screening of 96-well plates with 2 µl drops, a zoom setting of ∼2.3× works well. At this magnification the field of view captures the entire width of the drop, but the top and bottom edges are cut off. Images of a typical 2 µl drop at various magnifications are shown in Supplementary Fig. S1.

We also experimented with a recently published, neural-net-based, automated crystal-recognition algorithm (Bruno *et al.*, 2018 ▸), which did not perform as well as we had hoped. The algorithm was trained on a very large data set of crystallization droplets, but the size of the drops and the number of pixels in the images were generally smaller than those produced by the system described here. A machine-learning system trained to recognize crystals in images more similar to those produced by this system would likely yield more accurate interpretations of the crystallization experiments.

To further illustrate the general utility of this device, we used it to take brief movie clips of *Caenorhabditis elegans* (see supporting information), and images of worms in a 12-well plate using a Four Thirds format camera at minimum magnification and using the Amscope camera at maximum magnification are shown in Supplementary Figs. S2(*a*) and S2(*b*). The system can also be used to image cells in suspension or attached to the bottom of the plate, as is common is cell-screening procedures (Supplementary Figs. S2*c* and S2*d*).

## 6. Concluding comments   

The imaging system described here was assembled at a fraction of the cost of a commercial equivalent. We were pleasantly surprised to discover that relatively inexpensive motors and other moving parts yielded an imaging system that is precise enough for crystal imaging and robust enough for daily use. In addition, the small footprint of this system allows it to function in a tall glove box. A system of this sort is, however, unlikely to be satisfactory for all users. This system images one plate at a time, and those with very high volume needs will be better served by a setup that allows manipulation of multiple plates and automated imaging at programmed intervals. In addition, some may find the construction process difficult, as care must be taken to ensure that the parts are tightly screwed, soldered and glued, and that all axes move smoothly.

In keeping with the long tradition of sharing software and methods in the crystallographic community, the plans and software associated with this project are all being made freely available. They are included in the supporting information to this paper, and updates to the hardware, software and procedures will be available at https://abohm01.pages.tufts.edu/. Users are encouraged to make modifications to these as they see fit. One can easily envision a variety of refinements, additions and improvements to the device and methods described here. A graphical user interface specifically dedicated to imaging multi-well plates, for instance, would make it easier for the uninitiated to use this technology and alleviate the need for the separate Python script that writes the G-code files, image processing could be used to automatically align the device, an inexpensive light source for the imaging of fluorescence-labeled proteins could be added (Tarver & Pusey, 2017 ▸), an automated crystal-identification system could be incorporated and, with a larger area of movement and a redesigned plate holder, a small ‘hotel’ could be built into the device so that multiple plates could be imaged. We hope that these advances will also be freely shared.

## Supplementary Material

Plans and software associated with this project.. DOI: 10.1107/S2053230X18016515/pg5077sup1.gz

## Figures and Tables

**Table 1:** Cost of materials

Item	Cost (US$)	Source
Three-axis CNC router/carving machine (model 1610)	179	Ebay merchant†
Microscope focusing rack (model RF-A70)	96	https://www.amscope.com/
1080p HDMI digital camera (model HD200VP-UM)	430	https://www.amscope.com/
1 inch conduit, 1 inch floor flange and connector	16	Home Depot
1/4 inch clear acrylic (17 × 20 cm)	20	Home Depot
3/4 inch plywood base (1 foot wide shelving will work)	8	Home Depot
1 W pure white, 12 V LED (Philmore ID 11-2900)	10	http://www.youdoitelectronics.com/
12 V relay (Omron G5LE-14 12VDC)	2	https://www.mouser.com/
PLA for 3D printing	20	http://www.3dsolutech.com/
HDMI monitor (1920 × 1080 pixels)	150	https://www.microcenter.com/
12× zoom lens with fine focus (MVL12X12Z)	1878	https://www.thorlabs.com/
1× extension tube (MVL10A)	543	https://www.thorlabs.com/
C-mount adapter (MVLCMC)	75	https://www.thorlabs.com/

† The model 1610 CNC machine was purchased new. It is available through a variety of Ebay merchants and is not branded.
