# Supplementary material for: An inexpensive system for imaging the contents of multi-well plates
Source: Acta Crystallogr F Struct Biol Commun. 2018 Nov 29;74(Pt 12):797–802. doi: 10.1107/S2053230X18016515 (PMC6277959; doi:10.1107/S2053230X18016515)

Figure S1

Crystal-containing drop at various magnifications. All images were taken using the “Amscope camera.” The drop had a volume of 2 microliters when the crystallization experiment began, and volume was likely reduced by vapor diffusion to aproximately 1 microlter at the time of imaging.

The individual crystals within the clusters are approximately 5 microns wide and 40 microns long. The images have not been enhanced or altered in any way.

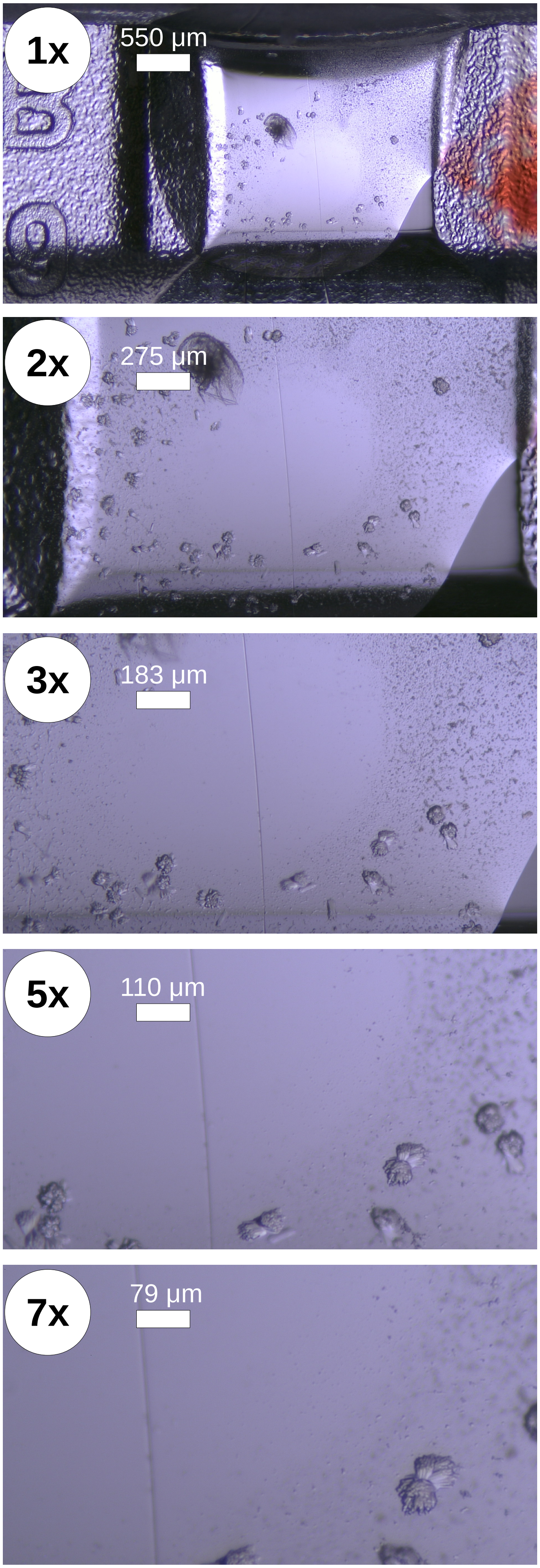

Figure S2

Other biological samples.

A. The entire well (25 mm across) of a 12-well plate can be seen using a micro 4/3 format camera and a zoom setting of 0.58 x. (Note the vignetting at the corners.) An adult *C. elegans* worm can be seen near the center of the field. A conventional microscope light source was used to illuminate the large area.

B. At 7x magnification features within the worm shown above as well as eggs (near the bottom of the image) are clearly visible. This image was taken using the smaller format “Amscope” camera. The detector (5.5 x 3.4 mm) is 3.8 times smaller in each dimension than the camera used in part A.

C. Jurkat cells, which grow in suspension, as seen at 5x magnification with the smaller format camera. This image was taken from the top, and through the plastic dish cover. The image would be clearer if taken from the bottom.

D. Fibroblasts (adherent cells), as seen at 7x magnification with the smaller format camera. Again, we expect the image would be clearer if it was taken from the bottom side and not through the top of the plastic cover.

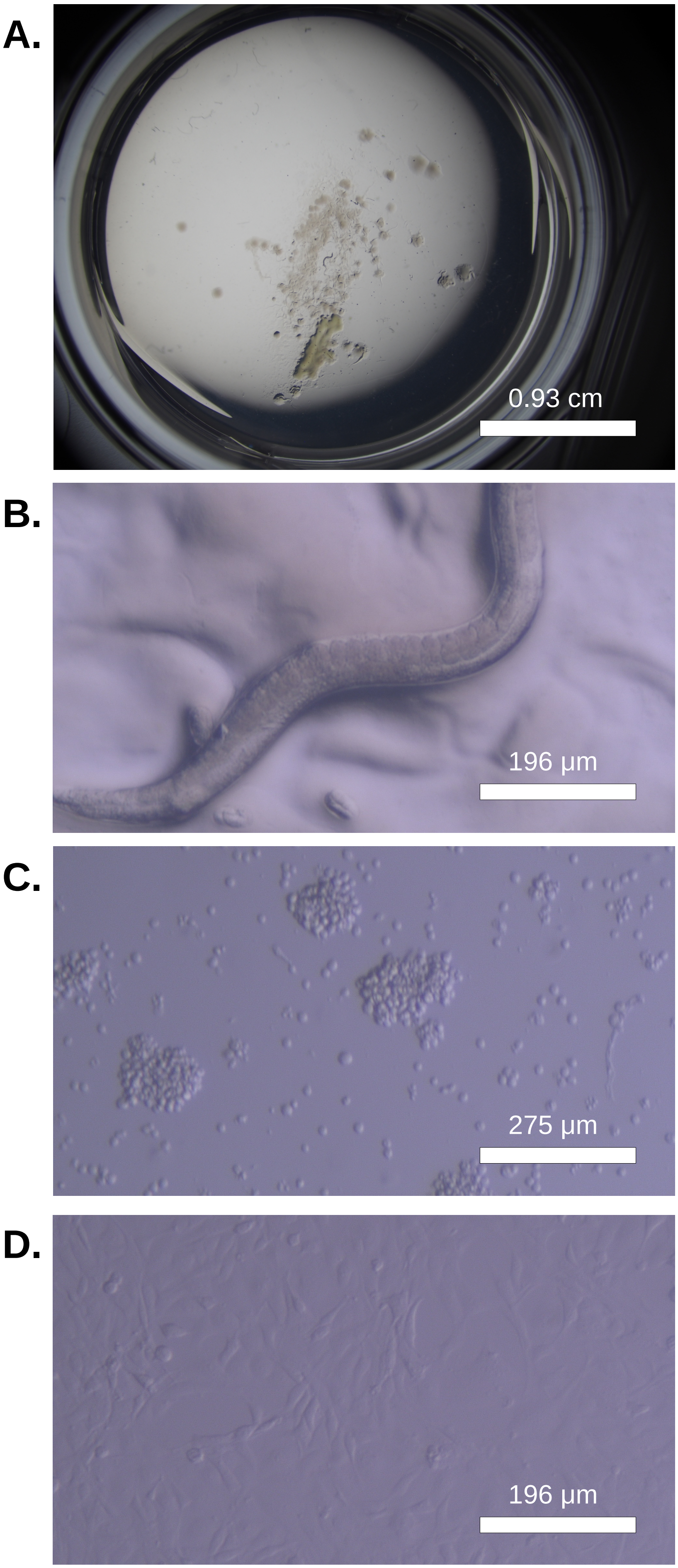

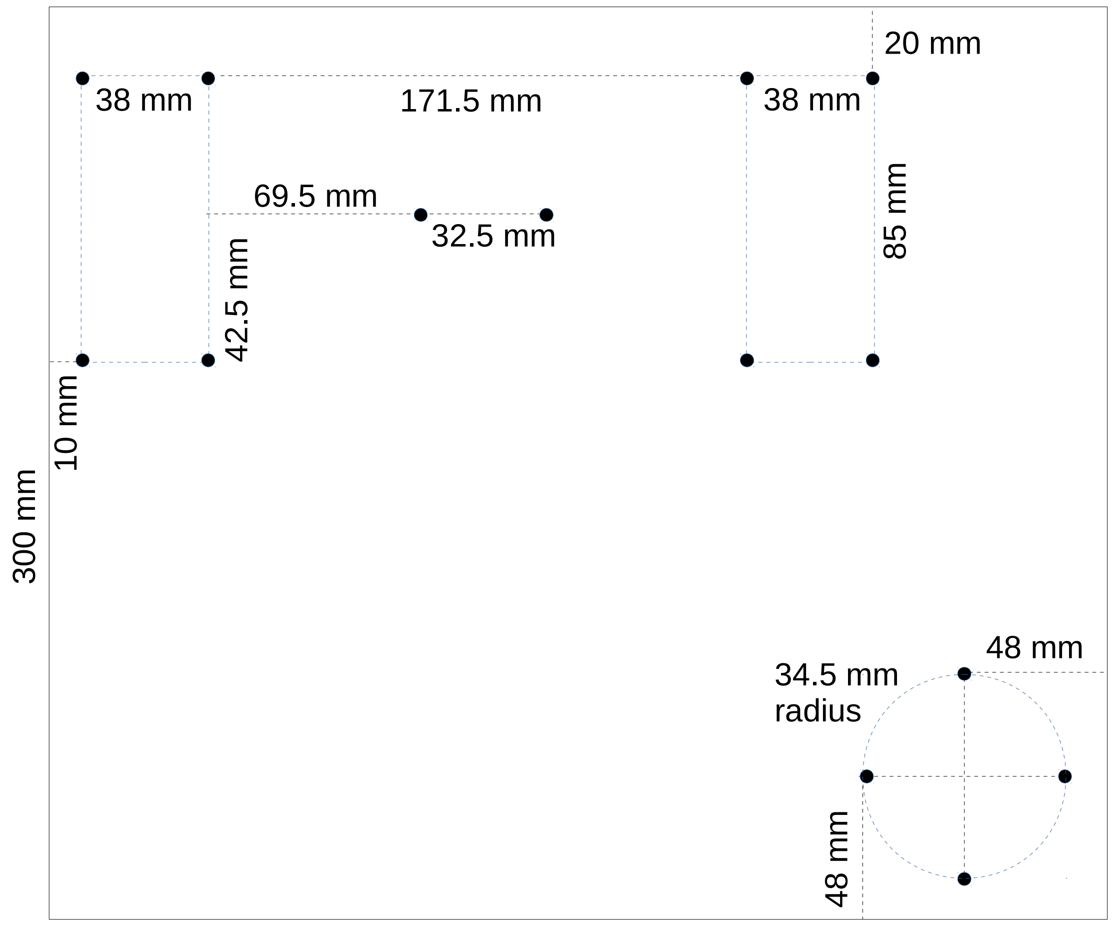

Figure S3

A. Base (above). The base is made of 3/4" plywood. (A piece of laminated shelving was used.) Holes at the indicated positions attach the translation stage and the base of the microscope post.

B. Sample holder. The sample holder is made of ¼ inch thick clear acrylic. Corner 1 should be glued with acetone at the position shown, flush to the right edge. The round piece that attaches the sample holder in place of the cutting tool is attached via the hole near the top. after tightening the bolt, it should also be glued with acetone.

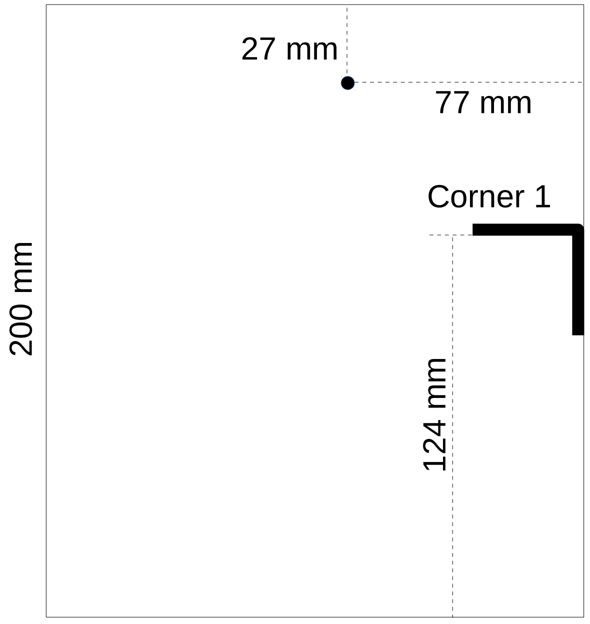

Figure S4

Connections for computer control of the left mouse button. The black mouse wire has a standard USB connector which is not altered. The grey wire is added. It runs from the terminals of the left mouse button click switch to the relay attached to the CNC control board.

Control board

Tool power (12V) switched by G-code m3 (on), m4 (off)

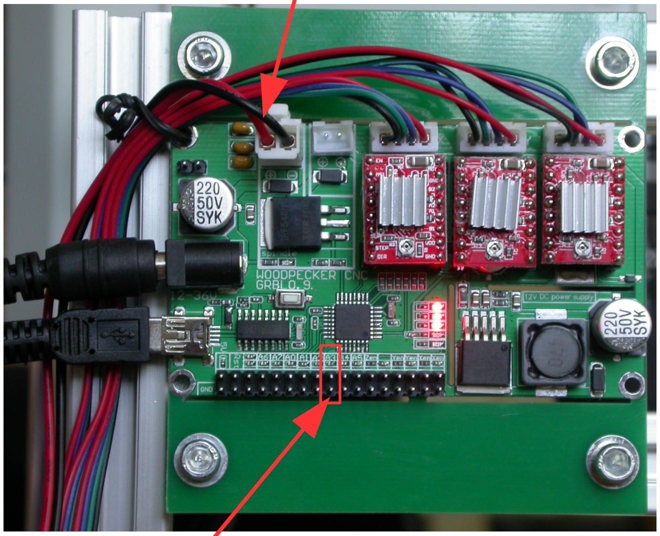

Header A3 (5V).
Switched by G-code m9 (on), m8 (off)

Relay

Omron G5LE-14 12VDC relay*
connections (bottom view)

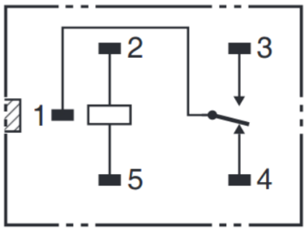

Pins 1 and 3 go to the new mouse wires.
Pin 2 goes to 12V ground (black wire)
Pin 5 goes to 12V power (red wire)
Pin 4 is not connected

*other 12V relays should work equally well.

Mouse

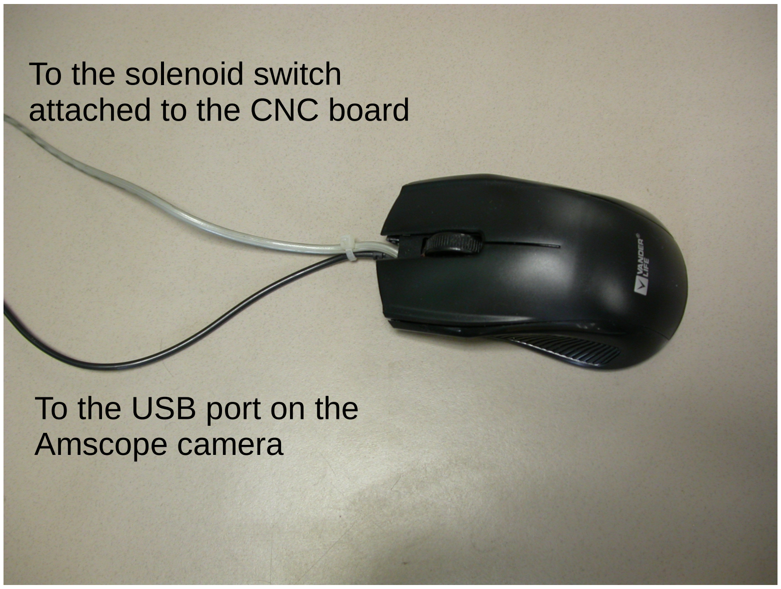

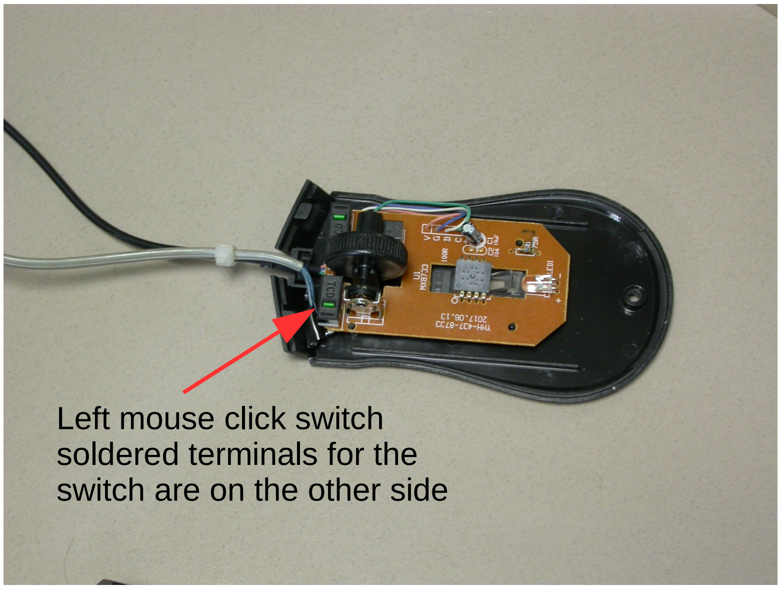

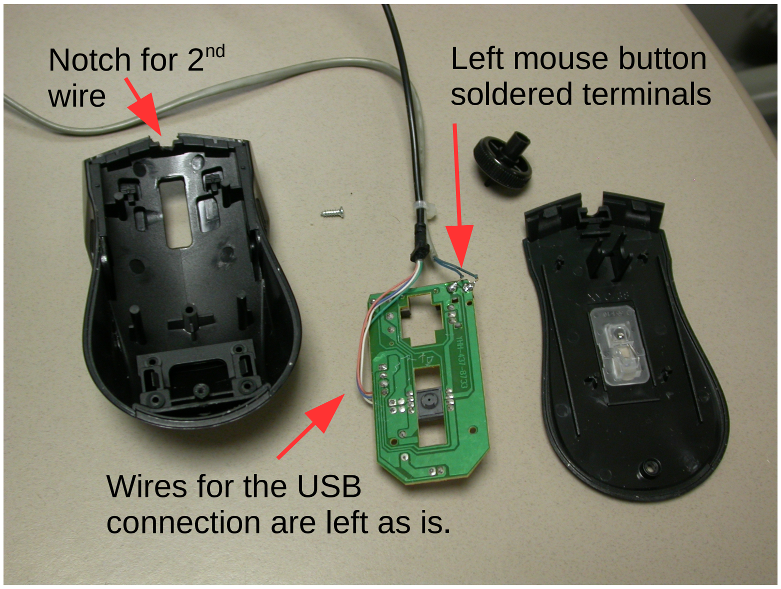

Supplement: Supplementary file 1 [file f-74-00797-sup1.gz › supplement/revised_supplementary_figs.pdf]
