# Supplementary material for: An inexpensive system for imaging the contents of multi-well plates
Source: Acta Crystallogr F Struct Biol Commun. 2018 Nov 29;74(Pt 12):797–802. doi: 10.1107/S2053230X18016515 (PMC6277959; doi:10.1107/S2053230X18016515)
Supplement: Supplementary file 1 [file f-74-00797-sup1.gz › supplement/revised_supplementary_text.pdf]

Supplementary materials.

<u>Python scripts and configuration files</u>

plate_scan.py – writes a G-code file that controls movements of the plate and the camera shutter during image acquisition

plate_scan.config – a sample configuration file for plate_scan.py

prep_stack.py - writes a unix script that processes z-staked images creating a single image with enhanced depth of field.

prep_stack.config – sample configuration file for prep_stack.py

<u>3D-printer files</u> (in each case, editable FreeCAD files are also included)

scope_pole2.stl: Adapter piece to fit the microscope focusing rack snugly on the steel conduit which supports it.

fan_holder.stl: Holds the small fan (40mm wide) that circulates air beneath the sample. You will not need the fan unless you are using a more intense light than the one described.

lh1_a.stl, lh1_b.stl, lh1c.stl: Adjustable base pieces for the LED light source.

corner1.stl, corner2.stl: Corner 1 is glued to the acrylic with acetone. Corner 2 should be printed twice, and three magnets should be glued into each hole. It best to print all corner pieces at 0.1 mm resolution. Sand them smooth if the print does not yield clean surfaces.

plate_holder3.stl: This should be bolted and glued (with acetone) to the acrylic plate holder.

mic_adapt2.stl: This adaptor piece goes between the microscope lens and the focusing rack.

Construction notes:

Assembling the translation stage:

The model 1610 CNC engraving machine kit contains all of the parts needed for the frame. It also contains the motors, translation screws, bearings, and the "Woodpecker" control board that will drive the device. Images of the device as originally designed and of the completed automated microscope are shown in **Figure 1**. First assemble the bottom frame following the instructions included with the 1610 kit. Next, attach the rod holders for the Y-axis translation to the bottom side of the frame (not the top side). These should be placed as far to the sides as possible, with one mounting screw on the front (or back) part of the frame and the other screw on the side part of the frame. Now assemble the gantry following the instructions, but attach it to the bottom frame such that the the metal corner bracket and the 3D printed angle braket are on the back side (not the front). Mount the X and Z translation parts as close to the bottom (not at the top) of the gantry as possible. Now attach the rods with the four sets of bearings at the bottom of the machine. Also attach the screw, motor, and the bearing for the Y translation to the bottom side of the bottom frame. The screw should run from the center of the back to the center of the front. It will probably help to turn the machine on its back when doing this last part. There is no harm in turning the translation screws by hand with the motors attached. You'll want to bring the respective parts to the ends of their translations before tightening each side up. When you are done, all three axes should move without any significant resistance. If you do feel resistance, loosen up the rod holders and/or the parts holding the motors to relieve the stress before tightening them up again.

Microscope post:

The microscope post is made from an 18 inch piece of 1" steel conduit, the type used to hold electrical wires. (You cannot use black pipe because the outer diameter is too large.) This is attached to a 1" floor flange (plumbing materials). To connect the two, use a threaded steel connector normally used between conduit and an electrical box. Screw the connector into the floor flange tightly, then solder

(with a torch, not a soldering iron) the conduit to the connector. If you cannot find the connector piece, it is possible to screw the unthreaded conduit into the flange (some force will be required).

Mounting the translation stage and microscope post to the base:

We initially used a piece of half inch thick aluminum for the base, but then found that a sturdy piece of flat ¾ inch plywood or laminated particle board works just as well. Wood is less expensive, easier to find, and far easier to cut and drill. Attaching rubber feet to the corners will ensure that the wood stays dry and, by raising the base about a half inch, the rubber feet alleviate the need to countersink the 14 screws that attach the translation stage and microscope post to the base. The dimensions of the plywood and the location of these holes is shown in **Figure S3** below. The positioning of the screws is important for smooth movement. It's best to make the holes a bit larger than necessary so there is scope for minor adjustments. The stage and post should be mounted with 1 ¼ inch long screws with washers on the plywood side. These screws should be snug, but not so tight that the plywood deforms. Once the translation stage is attached, mount the microscope pole to the base in the same way.

Electrical connections:

With the translation stage mounted to base, attach the circuit board with screws from the CNC kit to the back side of the gantry and then connect the motors to the circuit board following the CNC kit directions. You will not be using the cutting tool that came with the kit, but you do need the two-stranded wire that connects it to the circuit board. Solder the ends of the wire that would have gone to the cutting tool to the a 12V relay (**Figure S4**). Now solder pin connectors to the two relay leads that will be connected when the switch is energized. These pins will connect to the wire that controls the camera shutter.

If you are using a mouse-controlled Amscope camera, you will need to add an extra two-stranded wire from the mouse to the solenoid. Open a USB mouse (it does not need to be the one that came with the camera) and find the switch connected to the left mouse button. Carefully solder the new wires to the leads of this switch as shown in **Figure S4**. Then put a cable tie around the two wires

leaving the mouse so that the newly soldered connection is not accidentally pulled loose. You may need to make a small notch in the top of the mouse to allow space for the new wire to exit the mouse before you put the mouse back together. The USB port of the mouse gets plugged into the camera. The other ends of the new mouse wires attach to the solenoid. When you are done, you'll be able to click the left mouse button either with the normal mouse button or by entering G-code commands m3 (equivalent to pushing down the mouse button) and m5 (equivalent to letting the mouse button back up). Other cameras may have more elegant means of remotely triggering a photo or video.

Should you need a second computer-controlled switch, you can connect a 5V solenoid to the connectors on the Woodpecker board that are marked A3. The 5V power across these pins is controlled by G-code commands m9 and m8.

Connecting the microscope and camera:

Two 3D-printed adapter pieces were used in the microscope assembly. The radius of the post is about .7 mm narrower than that of the hole in a the standard microscope rack. Thus, a 3D printed piece was designed to take up the extra space. This will need to be curled somewhat to get it inside the focusing rack. A second 3D-printed adapter goes between the microscope optics and the large hole in the focusing rack. You'll need to bend it a little to get it around the lens before it is placed into the focusing rack and tightened in place with the screws on the focusing rack. This adapter piece is off center, and it should be inserted such that lens is away from the microscope post. (The extra distance is needed when scanning 24 well plates.) Also, you'll want to have the marker for the zoom setting pointed towards the front of the system.

Light and fan:

Three 3D-printed pieces were designed for the light, and an additional piece was designed to hold the fan. Connect the two printed pieces of the light base with a 2.5" long screw and attach the LED light to the base with double-stick foam tape. A stack of 10 small polystyrene weigh boats (Fischer Scientific #08-732-112) are used to diffuse the light. The third printed piece of the light assembly sits over the

weigh boats and is purely cosmetic. Initially, we used a conventional microscope light source, and the fan was necessary to ensure that the sample was not heated. The fan is probably not needed with the 1W light. Should you want it, connect a 40mm wide computer fan to the printed fan holder. Then connect the fan to a 12 V power source. The fan draws very little current, and it can be wired in parallel with a 12V light source.

<u>Sample holder</u>

The part that holds the multi-well plate is made from a piece of clear, ¼ inch acrylic (**Figure S3b**). (This can be easily cut on a table saw with a sharp blade.) A 3D-printed piece with the same diameter as the cutting tool in the original CNC kit attaches the acrylic to the translation stage. This should be screwed to the acrylic and then glued with acetone. (With the parts screwed together, dispense the acetone to the joint with a small syringe. Capillary action will draw it in.) Now glue (again with acetone) the "top right" corner piece as shown in the figure . The opposite corner of the multi-well plate is held by a pair of magnetic brackets - one above the Plexiglas, the other below. Each hole in the 3D printed pieces fits three stacked neodymium ring magnets that are 0.375" in diameter. Be sure to orient the magnets so the pair of brackets adhere to each other in the proper orientation before gluing the brackets in place with a small amount of epoxy. The completed sample holder can now be fitted onto the translation stage; you will need to move the z axis near the top of its translation range to insert it. (It's okay to turn the screw by hand.) Align the acrylic sample holder such that the front is parallel to the bottom frame, and then tighten the screw that holds it in place well. Don't worry about getting the alignment perfect; the software calibration procedure will correct for small errors.

<u>Algorithm for calculating drop positions:</u>

The mathematical corrections based on the top left (tl), bottom right (br) and bottom left (bl) coordinates after zeroing the machine at the top right sample are summarized in this section. This

algorithm is implemented in prep_stack.py, and the procedure for obtaining the coordinates of the corner drops is outlined in the calibration section below.

First define a matrix that applies a linear correction – this assumes the plate is flat and x,y,z translations are all perfectly orthogonal. (n_x and n_y are the number of columns and rows on the multi-well plate.)

$$\text{linear correction matrix} = \begin{bmatrix} \frac{tl_x}{(n_x-1)} & \frac{br_x}{(n_y-1)} & 0 \\ \frac{tl_y}{(nx-1)} & \frac{br_y}{(n_y-1)} & 0 \\ \frac{tl_z}{(nx-1)} & \frac{br_z}{(ny-1)} & 0 \end{bmatrix}$$

This matrix is first used to calculate the predicted position of the bottom left sample (bl_{linear}).

$$\vec{bl}_{linear} = [\text{ linear correction matrix }]\ [(nx-1),(ny-1),0]$$

Next, the correction for x, y and z are caculated using the actual position ($bl_{measured}$):

$$\text{non-linear correction vector} = \frac{\vec{bl}_{linear} - \vec{bl}_{measured}}{(nx-1)(ny-1)}$$

The position of each sample is calculated by applying the linear correction matrix and then applying the non-linear correction factor. For instance, the drop on the first row in x and the 3rd row in y has coordinates [0,2,0] (z is always zero at this point), and the x,y,z coordinates of this sample in mm is given by:

$$\text{position} = ([\text{linear correction matrix}]\ [\ 0{,}2{,}0\ ])\ [\text{non-linear correction vector}\ ]$$

Olympus camera notes:

As mentioned in the main text, micro 4/3 format cameras have a much larger detector (21.6 mm x 13 mm) than that of the “Amscope camera” (5.5 mm x 3.4 mm) used to collect most of the images

discussed here. These larger-format cameras are not much more expensive and have many more pixels (i.e. 16 MP vs. 2 MP). Moreover, they are easily coupled to the lens described here via an inexpensive (~$10) C-mount adapter. At any magnification, a micro 4/3 format camera should deliver similar image quality, but with a 3.8x larger field of view both dimensions. As mentioned in the main text, however, the images we took at high magnification were marred by shutter-induced camera shake. We tried to circumvent this problem by adding an inexpensive LCD light valve which was powered by the 5V connections labeled A3 on the control board and controlled via G-code commands m9 and m8. The idea was to open the LCD shutter after the camera's mechanical shutter had opened and the shutter vibration stopped. This was successful, but the LCD was not optically clear, causing the image to deteriorate somewhat, and negating the benefit of the larger format detector. Thus, those choosing to build a system with a larger format detector will want both proportionately higher magnification optics and a camera with an electronic front curtain shutter to prevent camera shake. It is important to note that as the magnification increases, the depth of field decreases (see table below). This can be compensated for by taking more images at more closely spaced z-intervals, but the large number of large images will take significantly more time to process. Moreover, because HDMI computer monitors cannot display more than ~2 megapixels, the extra data will not be visible unless one digitally zooms in on the image. In short, though use of a micro 4/3 format, consumer-grade camera is feasible and the camera itself adds comparatively little to the total cost of the system, we anticipate that most users will ultimately be more satisfied with a smaller format detector.

Computers and software installation

We recommend using two computers, an inexpensive machine to collect the data (we use a 14 year old laptop), and a faster one to process and view the images. The images will be written to an SD card within the camera. The image dimensions are 1920 x 1080, and are best viewed on a 1080p

monitor. You will probably want one such monitor by the imaging system (for alignment and manual inspection of drops) and another at the computer used to view the final, digitally-enhanced images.

The circuit board that drives the CNC machine is an arduino-based device running GRBL. There is no need to update the version of GRBL on this board. The board is connected to the inexpensive (or old) computer running a G-code sending program via a USB cable. We recommend the open source program bCNC (https://github.com/vlachoudis/bCNC) because it has programmable buttons (discussed below). On linux, you may need the following packages: `apt-get install python-tk python-serial python-imaging-tk: python-opencv` On some linux systems you may also need to give yourself access to the USB port: `sudo usermod -a -G dialout $USER` Almost any computer with a usb port can be used to drive the translation stage, and the software runs on Linux, PC and Mac. We've also used the program Universal-G-Code-Sender (https://github.com/winder/Universal-G-Code-Sender) in place of bCNC. The python script plate_scan.py and sample configuration file (included here) should also be uploaded to this computer. This script writes the G-code that the computer will sent (though bCNC) to the imaging system.

The scripts and instructions provided here assume that the computer used to process the images is running Linux. They will need to be adapted if you choose to use a windows or apple system. To produce z-stacked images, you will need to install hugin (http://hugin.sourceforge.net/download/). Hugin contains the programs align-image-stack and enfuse, which will be used to create images with enhanced depth of field. To view the images, you will also need image-viewing software. We use Ristretto Image Viewer. Other such packages should work equally well, and they are normally installed by default on Linux systems.

Calibration and G-code generation:

The steps below detail the procedure for preparing a calibration file. A separate file will probably be needed for each type of multi-well plate that you scan. These calibration files are used to

generate the G-code files that drive the machine movements. In general, the calibration and G-code files only need to be updated if the alignment of the machine parts change. If the machine is driven beyond its physical limits or if the power or computer connection is unexpectedly interrupted, you will need to re-zero the system (step 1 below), but you will probably not need to create new G-code files.

1. Turn on and zero the system:

After starting bCNC, go to the “File” tab and select the correct USB port. Then click “Open” to connect to the arduino board. A short alarm normally sounds at this point. Now switch on the camera and the light source and go to the bCNC “Control” tab where you will find buttons for moving the stage. After placing the plate on the acrylic sample holder, slide the magnetic corner piece so the plate is securely held. (It’s best to slide the top and bottom parts of the corner piece at the same time.) Use the software to move the plate so that the top right drop is centered in the microscope view, and select the zoom setting that you will use to collect your data. It is okay to use the focusing rack and/or the focus ring on the lens at this time, but they should not be touched once the machine is zeroed. It is best to focus on an object at or near the bottom of the drop. To zero the system, click the buttons labeled x=0, y=0 and z=0. Positive movements in x move the plate to the right (so the drop at position A1 would eventually come under the scope). Positive movements in y move the plate towards the back (so row H comes under the microscope). Positive movements in z move the plate up towards the lens. (Note that if you begin with the bottom of the crystallization drop in focus and move the plate away from the lens (-z) you will see top of the drop come into focus).

2. Get coordinates of the corner drops:

With the system zeroed, use the bCNC buttons to move to the four corners of the plate and record the x,y,z coordinates of the top left, bottom right and bottom left drops when the drops are in focus. As before, it is best to set z such that the bottom of the drop is in focus. These coordinates must be typed into the configuration file discussed in step 4. The coordinates can also be input into bCNC’s programmable buttons. This will allow you to quickly check the alignment when scanning subsequent

plates. To program the buttons, right click the button and enter the g-code command to drive to the specified coordinate (i.e. `g0 x 98.87 y -1.52 z 0.42`).

<u>3. Choose the z-spacing and the number of images per drop:</u>

First move to a sample that contains precipitate (or crystals) near the the top and the bottom of the drop. Now, by moving the plate up and down in z with bCNC and examining the image on the monitor, determine the thickness of the drop. It is best to assume the drop is at least 50% thicker than it really is. This will ensure that the contents of the drop will be captured, even if the calibration is slightly off or if the focus is slightly distorted by the curvature of the liquid. The image alignment and depth of field enhancement works best if there are clear focused features in adjacent frames. Thus, the z-spacing should be <u>smaller</u> than the depth of field. You can use the following table to estimate the depth of field depending on the chosen zoom setting. These values will change if you use a different lens.

Magnification	Depth of field (estimated
0.58 x	1.4 mm
1 x	1.0 mm
2 x	0.45 mm
3 x	0.2 mm
5 x	0.07 mm
7 x	0.05 mm

For example: In 96 well plates with 2 microliter starting drops (1 uL protein + 1 uL well solution), the drops are initially about 0.6 mm thick. If we screen at 2.5x magnification, the depth of field is around 0.3. If we set the z-steps to 0.15, we need at least 4 images to capture the contents of the whole drop. To account for miscalibration and optical distortions, it's best to take 5 or 6 images. The python script will calculate the total thickness of the stack and then begin the stack of images at 30% of this thickness below the bottom of each droplet.

<u>4. prepare the calibration and G-code files:</u>

A default z-stack thickness and number of images must be written in the configuration file for each type of plate you scan. This should be written in plain text, and placed in the directory that contains the python script plate_scan.py. A sample calibration file for a 96 well plate is shown below.

```
12                  # drops in the x direction
8                   # drops in the y direction
98.82   -1.52  0.44 # coordinates of the top left drop
 0.69   62.92  0.53 # coordinates of the bottom right drop
99.47   61.04  1.10 # coordinates of the bottom left drop
0.15                # default spacing between image in z
5                   # default number of images per drop
```

To run the python script type: `python plate_scan.py config_file.txt output_file.gcode`

You may choose any names you like for the configuration files and gcode files, and you will most likely want to have different files for different types of plates. You do not need a different configuration file if you change the magnification, but you may want to adjust the z-spacing of images and the number of images per drop. The plate_scan.py script prompts you for such changes before the G-code is written.

<u>Scanning a crystallization plate:</u>

Begin by turning on the machine and zeroing the system on the top right drop as in step 1 above. If you've set up the programmable buttons, you can use these to check the alignment.

Now go to the "file" tab in bCNC and load the gcode file you prepared in step 4 above. Then move the mouse attached to the camera to the bottom of the window and delete any images on the SD card. (If there are many images, it may be faster to move the card to a computer.) Then move the mouse to the left side of the window and leave the pointer on the "snap" icon. Clicking the mouse here will take a picture – but don't take any pictures, the system will do this once the G-code program begins to run. To run the G-code, open the "control" tab in bCNC and click the arrow shaped play button. At this point you should see the machine move to drop A1 and begin to take images. It will take 10-15 minutes to image the entire plate.

Once all the images are taken, move the SD card from the camera to the computer on which you'll do the image processing and viewing. I recommend you create a new directory for each set of images and that you copy the prep_stack.py and prep_stack.config files into each such directory before you run them. The images will be moved from the location specified in the configuration file (usually this will be the SD card) to a subdirectory named `raw_images`. The prep_stack.py configuration file has the following format:

```
5                          # number of data images per well
96                         # number of wells on the plate
IMG                        # image file root
/media/user/SD_card/DCIM  # location of the image files
```

The python script is run with the command `python prep_stack.py config_file.txt output_file.com`

To process the images you'll need to run the newly created command file: `source output_file.com`

You can ignore warning messages about the lack of EXIF metadata in the image files.

The final, enhanced images are named `stacked00001.tif stacked00002.tif`, etc. When the command file finishes, you can view these with image viewing software such as Risteretto, Mirage, or Eye of Mate. It is best not to delete the original images until you have seen the stacked versions. To delete the original images: `rm -rf raw_images`

Good luck, and happy scanning!
